# Veterinary Ethics in Practice: Euthanasia Decision Making for Companion and Street Dogs in Istanbul

**DOI:** 10.3390/ani15172585

**Published:** 2025-09-03

**Authors:** Mine Yıldırım

**Affiliations:** Department of Common Courses, Kadir Has University, Cibali, 34083 Istanbul, Turkey; mine.yildirim@khas.edu.tr; Tel.: +90-212-533-65-32

**Keywords:** euthanasia, companion dogs, street dogs, veterinary ethics, end-of-life decision making

## Abstract

This article examines how veterinarians in Istanbul experience and navigate the ethical, emotional, and institutional complexities of performing euthanasia on dogs, with particular attention to the differences between companion and street dogs. Drawing on 29 in-depth interviews with private practice veterinarians in Istanbul, this study employs qualitative analysis using the NVivo 12 Plus software and reflexive thematic analysis to identify key patterns in moral reasoning, emotional labor, and clinical decision making. The findings indicate that euthanasia of companion dogs is often framed through shared decision making with guardians, emotional preparation, and post-procedural grief rituals. While still emotionally taxing, these cases are supported by relational presence and mutual acknowledgment. In contrast, euthanasia of street dogs frequently occurs in the absence of legal ownership, institutional accountability, or consistent caregiving, leaving veterinarians to bear the full moral and emotional weight of the decision. Participants described these cases as ethically distinct, marked by relational solitude, clinical ambiguity, and heightened moral distress. Six key themes that reveal how euthanasia becomes a site of both care and conflict when structural support is lacking are identified in this study, including emotional burden, ethical strain, and resistance to routinized killing. By foregrounding the roles of institutional absence and relational asymmetry in shaping end-of-life decisions, this study contributes to empirical veterinary ethics and calls for more contextually attuned ethical frameworks, particularly in urban settings with large populations of street dogs and culturally entrenched practices of collective guardianship and caregiving.

## Simple Summary

This article explores how veterinarians in Istanbul navigate the ethical, emotional, and institutional challenges of performing canine euthanasia, with a focus on the contrast between companion animals and street dogs. Drawing on 29 qualitative interviews with small animal practitioners in Istanbul, this study reveals that, while euthanasia of companion dogs often involves shared decision making, emotional preparation, and mutual acknowledgment with owners, euthanasia of street dogs typically unfolds in the absence of legal guardianship, institutional support, or shared responsibility. These conditions intensify the moral distress, emotional labor, and ethical strain experienced by veterinarians, who are left to carry the full burden of life-ending decisions alone. Through reflexive thematic analysis, six key themes are identified, including professional isolation, resistance to routinized killing, and the pursuit of dignity in death. This study contributes to empirical veterinary ethics by situating euthanasia as a relational and context-dependent practice, shaped as much by social and institutional absences as by clinical judgment. It calls for more nuanced ethical frameworks that acknowledge the uneven burdens faced by veterinarians—particularly in urban settings—where care infrastructures are fragile and the boundaries between medical action and moral responsibility are constantly negotiated.

## 1. Introduction

Canine euthanasia, professionally defined in guidelines as the technical termination of suffering, is commonly referred to as one of the most emotionally and ethically problematic parts of veterinary medicine and clinical practice. Veterinarians themselves have articulated the emotional burdens and ethical conflicts they endure while performing canine euthanasia, in the context of which they must navigate an ethical, professional, and emotional terrain where care and killing intersect in ways that are generally more complex than can be described. Euthanasia is not only a medical intervention or a unique performance on the margins of medical practice but also an ethical moment—one that stays with veterinarians, as has been reported in numerous qualitative studies, having far-reaching impacts on their mental well-being and health [1,2,3,4]. What has been called the “caring–killing paradox” [1,5] in the literature is something that many veterinarians do not experience as a theoretical idea but, instead, as an ethical dilemma and generative tension that lies at the very heart of their clinical practice.

For many veterinarians, even when euthanasia is ethically and medically justified, it does not seem to be morally conclusive. Decisions are generally motivated not by the animal’s status alone but also by several external factors—such as financial constraints [6], spatial limitations, resource deficits, the emotional needs of a sad owner or the demands of a bereaved animal lover [7], and the veterinarian’s inability to decide on the necessity or timing of euthanasia [8,9,10,11,12,13,14,15]. Most vets would like to facilitate a “good death” [16]—one that is painless, on time, and dignified, thus confirming the value of the animal and the moral integrity of the vet [17,18,19]. Under ideal circumstances, it might be what Quain [3] has termed as “the gift”: a terminal act of kindness granted to an animal who is about to die. Yet, for many veterinarians, ideals of euthanasia as a “good death” fail to materialize. Increased rates of moral distress, compassion fatigue [20,21], and emotional burnout have been linked with the chronic repetition of morally challenging situations prior to, during, and after euthanasia [10,22,23]. Particularly, when euthanasia is medically unjustified, delayed due to the client’s emotional needs, motivated by financial interests, or ethically questionable, its emotional impact can be devastating, leaving veterinarians with residual sadness, uncertainty, or a sense of unresolved conflict [24]. These feelings serve as risk factors for occupational mental disorders such as anxiety, exhaustion, compassion fatigue [25,26], clinical depression [27,28], and suicide [28,29,30,31,32].

The growing literature on euthanasia in veterinary ethics also generally revolves around companion animals, focusing on cases in which end-of-life decisions are typically made in consultation with caregivers after years of shared life. In such contexts, euthanasia—though emotionally painful—is often embedded in frameworks of mutual understanding, deliberative decision making, and farewell rituals that help to alleviate the emotional burden to a certain extent [8,9,10,14,25]. These processes, while difficult, create a shared moral terrain that can buffer veterinarians from the isolating effects of sole responsibility; however, such conditions are far from universal.

In Istanbul, where the mass depopulation of street dogs via euthanasia is illegal under the current legal framework, thousands of street dogs live outside the structures of formal ownership but within loose networks of voluntary caregiving and inter-communal guardianship; in this context, the ethical landscape shifts considerably. These animals frequently arrive at clinics alone, brought in by concerned citizens or volunteers who often withdraw once the prospect of euthanasia arises. In such cases, veterinarians are left to navigate complex moral decisions in the absence of relational or institutional accountability, assuming the sole responsibility for choices with significant ethical and emotional consequences [12,33]. Beyond psychological strain, many veterinarians describe a deep sense of loneliness surrounding euthanasia. This feeling becomes especially acute when the decision must be made in isolation, without the shared responsibility of an owner, a caregiver, or even a supportive colleague [34].

This absence of shared responsibility significantly amplifies what has been identified in the literature as moral distress—a psychological state arising when clinicians are unable to act according to their ethical judgment [10,13,35]. In the absence of the dog’s owner, a caregiver’s necessary and collaborative input, or definite institutional protocols, veterinarians are left to bear the full moral and emotional weight of life-ending decisions, imposing a burden that has been linked to burnout, compassion fatigue, and lingering emotional strain [10,14,33]. This strain is further intensified by the lack of collaborative dialogue and ethical or professional validation, either due to the complete absence of an owner or guardian, or to their presence but emotionally fraught decision making. Veterinarians often face distress when guardians withdraw from the process entirely or, conversely, when they insist on continued treatment despite poor prognosis and evident animal suffering. Financial limitations can prompt premature euthanasia, while emotional attachments may lead to prolonging treatment against clinical advice [6,12]. While some scholars have argued that strong human–animal bonds and the presence of a human owner/guardian in the process has negative consequences, a study in the U.S. has highlighted that many veterinarians feel more professionally successful when they give due consideration to the bonding between animals and their humans [36,37]. Veterinarians’ expectations and decisions to perform or resist performing so-called “convenience euthanasia” [38,39] creates a further ethical dilemma, while “behavioral euthanasia” [40] and its accompanying categorization of certain dogs as aggressive [41,42], “healthy but aggressive” [43], non-adoptable, or non-treatable [39,44] adds another layer of ethical complexity. As Bubeck et al. [45] noted, these dilemmas underscore how profoundly the relational context—whether defined by shared deliberation, emotional conflict, or professional isolation—all factor into a veterinarian’s experience of euthanasia of both companion and street dogs [19,33,34,46,47,48].

The work of euthanasia is not only emotionally taxing but also indicative of highly structural dynamics. Veterinarians providing medical treatment and care services for street dogs in Istanbul are often confronted with a fragmented system defined by unclear legal frameworks, restricted municipal budgets and, as well as changing beliefs, cultural attitudes, and public perceptions. Decisions about euthanasia in such situations are hardly ever a simple matter of medical prognosis and professional decision. Rather, euthanasia decisions about terminally ill, injured, or elderly street dogs often emerge from intersecting factors, including a lack of available space, financial resources, absence of long-term care availability, and the socially and legally marginalized status of street dogs themselves. These situations re-define the role of the veterinarian: from caregiver working in the interests of a bonded human–animal attachment, to medical subject navigating the deficiencies of animal care infrastructure.

In Turkey, animals are protected under the Animal Protection Law (Law No. 5199), originally published in the Official Gazette on 1 July 2004 [49]. The law aims to ensure that animals live a comfortable life, receive proper treatment, and are protected from pain, suffering, and all forms of cruelty. The 2021 amendment of The Animal Protection Law prohibited the routine euthanasia of healthy street dogs, prioritizing sterilization, vaccination, and return-to-origin practices instead. However, the recent 2024 amendments [50] have fundamentally altered this framework. Under the new provisions, street dogs collected by municipalities must be placed in shelters until they are adopted, and their transfer elsewhere or abandonment is explicitly prohibited. In addition, Article 5 of the amended law now permits the euthanasia of street dogs taken into shelters if they pose a danger to human or animal life and health, exhibit uncontrollable negative behaviors, have infectious or incurable diseases, and/or are legally defined as a “prohibited breed” [50].

The 2024 legislative amendments on the Animal Protection Law in Turkey have sparked widespread public protests and strong criticism from animal welfare advocates, legal experts, and professional associations, as they introduce broader discretionary powers without providing standardized clinical criteria for determining euthanasia [51,52,53,54]. The Turkish Veterinary Medical Association (TVHB) has publicly opposed the expansion of euthanasia practices for street dogs introduced by these amendments, emphasizing the need for preventive, humane, and scientifically grounded population management strategies instead. However, despite its opposition, it is crucial to note that The Turkish Veterinary Medical Association does not issue explicit, binding protocols on euthanasia decision making. This absence of standardized clinical criteria leaves municipal veterinarians, private practitioners, and shelter staff to interpret the ethics of euthanasia on a case-by-case basis, often under significant institutional and public pressures. Meanwhile, municipal shelters continue to operate under limited budgets, with inadequate veterinary staffing and significant disparities in resources across districts. Compared to international frameworks such as the AVMA [55] and WSAVA [56] Guidelines on the euthanasia of companion animals, persistent structural gaps are observed in the Turkish context, placing disproportionate ethical and emotional burdens on veterinarians who must navigate competing professional, institutional, and public expectations. While this study focuses on Istanbul, its findings may hold broader relevance for other metropolitan areas where municipal veterinary services manage free-roaming dog populations within fragmented institutional infrastructures of veterinary care and under limited professional guidelines. In such contexts, the absence of standardized euthanasia protocols, inconsistent sheltering practices, and an uneven distribution of municipal resources may similarly shape veterinarians’ ethical dilemmas and decision-making processes [57,58,59,60,61,62,63,64,65].

This article examines the ethical significance of structural and relational distinctions in veterinary euthanasia practice. Based on in-depth interviews with 29 small animal veterinarians in Istanbul, this study explores how end-of-life decision making is shaped by the status of the dog—as either a companion animal or a street dog—and the presence or absence of a shared relational framework. Utilizing reflexive thematic analysis supported by NVivo 12 Plus, the findings suggest that euthanasia should not be understood as a singular or inherently benevolent clinical act; rather, it emerges as a socially mediated and morally ambivalent practice, shaped as much by institutional absences and relational asymmetries as by medical judgment. By foregrounding the emotional, ethical, and institutional dynamics at play, this study contributes to the expanding field of empirical veterinary ethics. It underscores the relational context not only as a key determinant of how euthanasia is practiced and interpreted but also as a defining element in how suffering is distributed, affecting both the animals whose lives are ended and the veterinarians tasked with carrying out the act.

The discussion builds upon recent developments in empirical veterinary ethics that foreground the practical, emotional, and institutional contexts of ethical decision making around canine euthanasia. Christiansen et al. [14] have emphasized the importance of practical judgment and situated ethical reasoning within veterinary care. Likewise, Hartnack et al. [65] have highlighted the role conflicts that veterinarians face when navigating euthanasia, especially under conditions of institutional constraints. Emotional regulation and the emotional costs of pre- and post-euthanasia performances have also been explored by Hannah et al. [66], who underscored the need for veterinarians to emotionally detach and stay in their comfort zone. In line with Tannenbaum’s plea [67] for veterinary ethics based on human–companion animal interactions, this study addresses the need for imaginative and relational approaches in veterinary decision making. In light of empirical evidence, the present discussion contributes to these ongoing conversations by offering an empirically grounded analysis of how Turkish veterinarians experience and negotiate euthanasia decisions—particularly in cases involving street dogs, where relational absence and institutional gaps heighten their ethical complexity.

## 2. Materials and Methods

This study emerged from the recognition of a significant gap in the veterinary ethics literature concerning how euthanasia decisions are made in cases involving street dogs; namely, animals who may be socially visible and emotionally recognized within a community yet lack a singular legal guardian or proprietary claim. Unlike companion animals whose end-of-life decisions are typically made through formal owner–veterinarian relationships, street dogs exist within more ambiguous and diffuse frameworks of care and responsibility. This project was designed to examine how veterinarians working in Istanbul experience, interpret, and navigate euthanasia practices in such ethically and relationally complex contexts.

Much of the existing literature on veterinary euthanasia focuses on situations in which decision making unfolds within formalized human–animal relationships, typically involving a veterinarian and a legal owner. In companion animal practice, euthanasia decisions are understood as a shared, triadic process in which the veterinarian advises the owner, who retains legal consent and responsibility, on behalf of the animal patient [14,46,68]. In this context, studies have emphasized a collaborative dynamic: decision making around euthanasia is often shared but ultimately requires the explicit consent of the owner, framed by notions of animal welfare and professional authority [8,9,14]. Regarding the issue of companion animal euthanasia, scholarly attention has largely focused on the emotional, ethical, and communicative dimensions of this shared responsibility [3,4,47,69]. The dominant narrative portrays euthanasia as an ethically bounded interaction—veterinarians provide clinical guidance and emotional support, while owners navigate grief, regret, and surrogate decision making on behalf of a beloved pet. However, the contexts in which euthanasia is enacted outside this relational dynamic remain understudied, particularly in cases where no legally recognized guardian exists and the veterinarian must act alone or within diffuse communal frameworks.

In contrast to companion dogs, street dogs in Istanbul often exist within fluid, communal networks of care; they may be intermittently fed, sheltered, or medically supported by volunteers, neighbors, or activists, yet without a clear locus of responsibility. This study investigates how veterinarians make sense of their ethical obligations and professional authority in such cases, where responsibility is diffuse and medical decisions unfold outside the bounds of formal ownership. It also explores the emotional and moral tensions that arise in euthanasia cases—especially when the actions taken by the veterinarian provoke grief, guilt, or conflict in those who feel emotionally connected to the animal, despite lacking legal standing.

To investigate these questions, the current study carried out qualitative research underpinned by interpretive and ethnographic orientations. Between December 2024 and 29 April 2025 semi-structured in-depth interviews were carried out with Istanbul-based small animal veterinarians who all possessed experiential knowledge of conducting euthanasia on both companion dogs and street dogs. Recruitment was conducted through purposive and snowball sampling techniques in order gain participants of varied gender, age, years of clinical experience, and employment status (i.e., self-employed practitioners as well as those employed within private clinics or municipal veterinary services). As opposed to representativeness, this study aimed for richness and diversity of ethical thinking and emotional expression in end-of-life care, especially in situations characterized by relational uncertainty and institutional constraints.

Reflexive thematic analysis (RTA) was conducted, as outlined within the methodology created by Braun and Clarke [70,71,72,73]. RTA is an adaptive and responsive method for analysis which is particularly tailored to examine strongly affective and ethically charged material, especially in research that prioritizes participants’ meaning-making practice. The approach permitted situated and context-sensitive interactions with the data, in accordance with the ways in which the veterinarians’ decisions, doubts, and intuitions were presented, in conjunction with discrete institutional and relational arrangements [2]. NVivo 12 Plus was utilized for iterative coding, memo writing, and theme development, enhancing transparency and analytic depth.

Although the broader strategy for the analysis was guided by the tenets of RTA, some constructivist grounded theory strategies were also incorporated—specifically, in the initial stages of coding—as a way of remaining attentive to potentially significant patterns and conceptual linkages actively identified by the analyst [74]. This form of hybrid methodology employed has been shown to be effective in studies on veterinary ethics investigating moral ambiguity, emotional tension, and the mundane realities of clinical decision making [33,75,76,77,78].

This study is not claiming to generate generalizable findings; rather, it provides a grounded, context-specific description of how veterinarians conceptualize and enact euthanasia in different structural, relational, and emotional contexts. Specific consideration is offered to street dogs—animals with no formal owners that are characterized by certain care arrangements by the local volunteers, daily practices of sheltering, feeding and looking after. Through recording these observations, this study contributes to the new empirical corpus of veterinary ethics by shedding light upon morally important institutions that have heretofore largely been ignored.

Interviews with veterinarians reveal that end-of-life decisions are rarely shaped by clinical factors alone. Instead, they are deeply influenced by the presence or absence of relational bonds—between the veterinarian, dog, and its owner or caregiver—as well as the availability of clinical resources and institutional support. The present study seeks not to resolve these tensions but instead to bring them into view, in order to better understand their moral and emotional implications. By situating euthanasia within the everyday practice of veterinary care, this research foregrounds the ethical and affective labor involved in decisions surrounding street dogs—especially by contrasting this scenario with the more structured and relationally anchored euthanasia of companion animals. In doing so, this study underscores how the moral universe of euthanasia is differentially constituted yet remains a largely overlooked dimension in clinical and ethical debates.

### 2.1. Methodological Approach

Qualitative methods were selected due to their being particularly well-suited to investigating under-researched areas [79] and allowing for closer inspection of veterinarians’ emotions, ethical decision making, and attitudes towards euthanasia [3,10,80,81]. As minimal research has focused on veterinarians’ experiences with street dog euthanasia, this enabled a detailed investigation of how practitioners negotiate and make sense of this ethically difficult aspect of their work, thus enabling an understanding of the subjective, relational, and institutional contexts in which such decisions are made [82,83].

This research was epistemologically rooted in constructivism, with the understanding that knowledge is co-constructed between the researcher and participant and that veterinarians’ narratives represent their situated moral worlds [74]. As a researcher situated within long-term multispecies ethnography in Istanbul, the author recognizes that their positionality- working alongside animal care and rescue professionals—influenced the questions they asked and the meanings they interpreted as suggested by Lu et al. [84].

To this end, twenty-nine (29) qualitative interviews were held in an iterative process with veterinarians working in private clinics in Istanbul, who were chosen via purposive sampling to guarantee that the participants had first-hand experience of performing euthanasia on both companion and street dogs.

Interviews were semi-structured and guided by open-ended prompts. Each conversation fit the description of a “conversation with a purpose,” allowing veterinarians to reflect on and articulate their experiences in their own terms. The flexible structure of the interviews enabled a deeper exploration of participants’ perceptions, while also making room for follow-up questions that responded to the themes and patterns identified during ongoing analysis. The open-ended nature of the prompts encouraged the interviewees to share varied accounts of what it means to perform euthanasia work in veterinary medicine, with narratives ranging from intensely personal to institutionally constrained. Participants often traced their own biographical trajectories—shifting between sectors, negotiating institutional demands, or reflecting on how their ethics had been shaped over time. These conversations did not simply aim to obtain responses to pre-determined questions; the interviewees were allowed to define what mattered and what did not, setting their own limits on what could be shared.

The interview process and its ongoing revision were shaped by principles of constructivist grounded theory [74,84,85], which treats data collection and analysis as a reflexive, co-constructed process rather than a neutral extraction of facts. Instead of coding data against a fixed theoretical framework, patterns and categories were identified in the accounts throughout the analytic process, emphasizing meaning-making and context over categorization. The interview guide was revised iteratively as the interviews unfolded, with attention paid to newly emerging tensions, silences, or unexpected reframing in participants’ narratives. This approach foregrounded empirical openness, participant agency, and the belief that both the researcher and participant bring interpretive frames to the encounter. Constructivist grounded theory provided a flexible yet rigorous scaffold for attending to the emotional and ethical texture of euthanasia practices, especially as they unfold in settings where institutional clarity and relational certainty are often lacking.

Data collection and analysis proceeded concurrently, with arising knowledge informing later interviews—a hallmark of theoretical sampling in constructivist grounded theory-based methods, with insights generated through the analytic process informing subsequent interviews [74,84,85]. This allowed not only for flexibility but also for the further investigation of meaningful patterns as they emerged from the stories. Researcher reflexivity was upheld throughout, in order to remain sensitive to how the researcher’s position and assumptions influenced the collection and interpretation of data [86]. Ethical approval was granted before data collection commenced, and informed consent was gained from all participants.

### 2.2. Sampling

This study is based on qualitative interviews held with 29 licensed veterinarians based in Istanbul, Turkey. All participants were trained in small animal medicine and had substantial professional experience in the clinical treatment of dogs, including sterilization, surgeries, elderly care, cancer treatment, intensive care, and performing euthanasia. While several participants reported providing palliative or hospice services as part of their routine practice, only a few had completed formal in-service training, with just two holding additional certifications in end-of-life (EoL) care for companion animals.

A total of 29 veterinarians (coded VET01–VET29) participated in this study. These participants varied in terms of gender, years of professional experience, type of veterinary practice, and involvement with street dog euthanasia. Table 1 provides a summary of the participant’s characteristics, illustrating the diversity and informative power of the sample.

The participants were chosen according to the following specific criteria: each one needed to have at least six months of clinical practice and should have performed euthanasia on at least one companion dog and one street dog. These criteria were intended to guarantee that all interviewees had sufficient clinical engagement with the ethical, professional, and emotional facets of euthanasia regarding both companion dog and street dogs. This form of sampling, which was performed with the intent to achieve a specific purpose, is a hallmark of qualitative research [85,87]. As is customary in empirical ethics research, the methodology was designed to target veterinarians who could provide rational, experienced-derived reflections on the morally intricate processes underpinning the morally intricate decisions and actions assessed in this study [78,88,89]. This also follows the empirical boundaries of veterinary ethics, with the idea that participants be chosen not simply because of their professional title but due to their ability to appreciate the complex networks of relations, emotions, and institutions in the context of clinical practice, which bear both ethical and moral weight [3,90].

The sample consisted of 17 male and 12 female veterinarians, representing a range of professional seniority—from early-career practitioners with fewer than 5 years of experience to those with over 25 years in the field. Of the 29 participants, 18 were self-employed and 11 were employed in veterinary clinics or animal hospitals. All employed veterinarians had prior experience in independent veterinary practice, offering a layered view of euthanasia practices across professional settings.

To ensure access to rich experiences, varied approaches, and relevant accounts of veterinarians, a multi-pronged sampling strategy was employed. This included purposive sampling, which was performed to select information-rich cases with direct relevance to the research question, and snowball sampling, which relied on referrals through professional networks and veterinary associations. In a few cases, participants were identified through targeted web searches of veterinary clinics and institutional affiliations. This combined approach allowed for the inclusion of veterinarians working in diverse institutional contexts and brought heterogeneity to the sample in terms of gender identity, career stage, and veterinary practice. Such variation enriched the dataset and supported a more detailed understanding of how euthanasia practices—and the associated ethical and emotional labor—are shaped by contrasting professional trajectories [88].

While the participants’ demographic information, including gender, years of experience, and type of practice, was recorded to contextualize the sample, gender was not treated as a pre-determined analytical variable in this study. The primary objective was to explore veterinarians’ ethical reasoning, institutional pressures, and emotional dynamics around euthanasia decision making, rather than to test hypotheses about gendered differences. In line with the principles of reflexive thematic analysis [70,71,72], the analytical process was designed to allow themes to emerge inductively from participants’ narratives without imposing pre-defined categorical frameworks. Accordingly, no stratified or hypothesis-driven analyses based on gender were conducted.

### 2.3. Data Collection and Confidentiality

Data were collected through face-to-face, semi-structured interviews conducted in Turkish between December 2024 and April 2025. Interviews lasted between 50 min and 2 h and were audio-recorded with the participants’ consent. All interviews were transcribed verbatim.

The interviews followed a semi-structured format that balanced consistency with openness, allowing each conversation to unfold organically while attending to core thematic concerns. Six open-ended questions were developed to prompt reflective, emotionally grounded accounts of euthanasia practices. In the first question, participants were asked to describe what it feels like to perform euthanasia on dogs and how this aspect of their work has influenced their emotional well-being, ethical perspective, and broader approach to veterinary practice. The second question invited narratives regarding particularly difficult or emotionally charged cases, and how these moments had shaped their understanding of professional responsibility and ethical complexity. The third question focused on decision-making criteria, asking what factors participants weigh most heavily—such as clinical indicators, prognosis, treatment options, or input from guardians, caregivers, or institutions—and how these elements interact in practice. The fourth question explored the perceived differences between performing euthanasia on street dogs versus companion dogs, and how these contrasting contexts affect the ethical and emotional dimensions of their role. The fifth question addressed the emotional labor involved in navigating the responses of dog owners, informal caregivers, or community members during euthanasia, particularly in moments of grief, conflict, or ambivalence. Finally, the sixth question asked about the participants’ visions for a more ethical, humane, or animal rights-oriented approach to end-of-life care, especially for dogs without formal guardianship. These questions were not designed to elicit fixed answers but to open space for biographical reflection, ethical reasoning, and emotional depth when recounting the layered realities of veterinary euthanasia not just as a medical procedure but as an emotionally charged and ethically weighty part of their work.

Participants were first invited to reflect on how their experience with euthanasia has shaped them over time; for example, how it affects their emotional well-being, how it sits within their everyday practice, and what it demands of them as both clinicians and caregivers. Many spoke about specific cases that had stayed with them, including moments of doubt, regret, or quiet conviction, and how these moments had altered their sense of professional identity.

As the conversations deepened, the questions turned toward the decisions behind euthanasia: what factors come into play, from the dog’s condition and prognosis to the presence—or absence—of a guardian or caregiver. These stories revealed how practical constraints and relational dynamics often collide, making each decision more than a matter of clinical judgment. Participants were also asked to reflect on the emotional responses of those who bring dogs in for euthanasia—the tears, the silences, the second thoughts—and how they, as veterinarians, carry these emotions long after the procedure is over.

A central focus of the interviews was the difference in how euthanasia is experienced when the dog is a street animal, rather than a companion with a known guardian. These accounts shed light on what it feels like to act alone in morally uncertain situations, when no one else is there to share responsibility, grieve, or witness the moment. In closing, veterinarians were asked what they believe ethical euthanasia should look like, and what kind of care (i.e., emotional, institutional, and professional) needs to be in place to support it. Their responses pointed to a practice shaped not only by medicine or law but by the fragile—and often invisible—emotional labor it demands.

The open-ended format enabled participants to describe their experiences in their own words, bringing out contradictions, hesitations, and affective registers that more structured instruments might have missed. Semi-structured interviews and the principle of saturation are well established in qualitative veterinary ethics research, as they allow for in-depth engagement with morally complex and emotionally charged topics such as euthanasia, animal suffering, and professional distress.

The interviews continued until no new codes or significant themes emerged from subsequent interviews. Following each interview, fieldnotes and ethnographic vignettes were written to record contextual impressions, emotional dynamics, and emergent patterns.

### 2.4. Data Analysis

Reflexive thematic analysis (RTA) constituted the principal analytic framework, complementing the interpretive orientation of this qualitative inquiry. Developed to explore how individuals construct meaning within socially situated and emotionally complex contexts, RTA facilitates the examination of how veterinarians articulate moral distress, institutional constraints, and the affective dimensions of care [24,27,28,29,30,31,32,91]. Its theoretical flexibility proved essential in uncovering how ethical dilemmas, personal convictions, and contextual pressures intersect in end-of-life decision making. Considering the emotionally charged and politically contested terrain of dog euthanasia—especially when involving animals without formal guardians—RTA provided a method for the analysis of not only recurring themes but also moral ambivalence and emotional nuances. This approach avoids reductionist categorization in favor of the iterative development of themes through a reflexive process, acknowledging the active interpretative roles that scholars play in shaping analyses [89,92,93].

Moreover, RTA’s emphasis on reflexivity—where the researcher’s positionality and interpretive choices are transparent and integral—made it particularly fitting for capturing veterinarians’ lived ethical reasoning and emotional burdens leading to depression, mental disorder, even suicide [27,28,29,91]. Rather than imposing rigid coding schemes or seeking inter-coder reliability, RTA supported nuanced engagement with the participants’ accounts, recognizing contradictions, ambiguities, and emotional texture as analytically vital rather than problematic. This methodological stance enabled a rich contextual understanding of how veterinary euthanasia is experienced not merely as a clinical act but as a socially embedded, morally complex practice shaped by care, constraint, and compassion.

For the initial coding, line-by-line analysis was employed to stay close to participants’ accounts, enabling both descriptive (semantic) and interpretive (latent) coding. The functionalities provided by NVivo (i.e., hierarchies of nodes, coding stripes, and query tools) allowed for iterative refinement, with codes potentially being combined, reassembled, or new codes added as patterns of ideas cohered [74,94]. Throughout the process, the researcher developed analytic memos that were directly linked to codes and data, recording comments, interpretive choices, and building thematic concepts and, thus, promoting transparency and positional awareness [93,95]. Through analysis via Braun and Clarke’s model, the process went through the stages of familiarization, initial coding, iterative development of themes, and theme refinement. Themes were interpretive formulations rather than summary labels, and attention was paid to the ways in which veterinarians negotiate tensions between authority, emotion, and moral labor in euthanasia work. Ethical dimensions, emotional resonance, and relational context were considered as conceptual organizing ideas, which were most evident when contrasting street dog versus companion dog euthanasia stories. The analytical focus was not just on text content but also on aspects of vocal intonation—for example, hesitation, tonal shifts, and affective language—which are considered ethically and analytically relevant in veterinary settings. The use of a reflexive, iterative, and interpretative methodology guarantees that ethical nuances and emotional work are brought to the fore of euthanasia practice research—not boiled down to typologies but instead made visible through lived experiences.

The English translation of the excerpts supplied by the participants was carried out solely by the author.

### 2.5. Ethical Considerations

Ethical approvals for this study were granted by Kadir Has University Ethics Committee (Protocol Codes E-82741295-600-92377 and E-82741295-600-123260).

To protect participant anonymity, all identifying information (names, veterinary clinic details, specific dates) have been removed or altered in this article. Given the sensitivity of the topic, participants were offered the option to withdraw at any point or to refrain from answering any question that felt distressing. Recognizing the emotional vulnerability elicited when discussing euthanasia practices, interviews were conducted with care and patience, often concluding with a debrief and a verbal invitation for participants to reflect on what the conversation brought up for them. Several participants expressed that the interview itself offered an opportunity for them to process a distressing experience and a rare space for professional reflection.

## 3. Results

This section presents key themes identified through the reflexive thematic analysis based on in-depth interviews with veterinarians who had performed euthanasia for street dogs as well as companion dogs in Istanbul. Using reflexive thematic analysis as a base framework, the findings reveal how participants expressed their ethical justification, emotional labor, and professional behavior toward euthanasia under different relational and organizational contexts. The viewpoints expressed by veterinarians regarding their role duties and professional responsibilities showed considerable variability based on whether the canine was a street dog or a household pet, whether a human guardian was present, and on systemic considerations relevant to a given circumstance. Rather than taking clear stands on whether euthanasia was justified, most respondents expressed nuanced, context-specific, and often mutually incompatible thinking about care, harm, need, and moral action.

The following thematic analysis is organized to illuminate key aspects of this emotional and ethical landscape, including the tension between professional beliefs and institutional constraints, ethical considerations related to long-term exposure to death, and a collective desire for professional environments that move toward more ethically aware and psychologically sustainable directions.

The respondents expressed a varied range of opinions about how their experiences with euthanasia affected their thinking with respect to veterinary practice, emotional health, and moral dilemmas [68,69,75]. Within reflexive thematic analysis, according to Braun and Clarke [70,71,72,73], it is critical to note that themes cannot simply be seen as simple reflections or questionnaire item labels. In fact, they represent products of the analysis which are created through interpretative encounters with data, not simply mirroring the subjects or topics under research. Themes should represent patterns of meaning across a data set and grant a level of interpretive understanding about the research questions, rather than simply categorizing replies.

### Themes Identified Through Reflexive Thematic Analysis

The six themes presented below are characteristic of the iterative and interpretive processes and, therefore, constitute a cumulative structure of knowledge regarding veterinarians’ experiences, reasoning, and affective responses to euthanasia. Instead of analyzing themes as fixed categories, they are treated as constellations of meaning—reflecting the fluid interplay between clinical judgments, moral deliberation, institutional constraints, and affective responses. All of the themes identify a specific, but complementary, aspect of the moral terrain encountered by veterinarians when practicing with companion and street dogs at the dying stage.

Theme 1, Shouldering the Burden—Euthanasia as a Moral Responsibility in Veterinary Practice, explores how veterinarians internalize euthanasia not merely as a medical procedure, but as a morally weighty act that profoundly shapes their professional identity and emotional world. Theme 2, Ethical Strain and the Emotional Costs of Adverse Euthanasia, traces how moral distress emerges when euthanasia is mandated by systemic pressures rather than clinical judgment, highlighting the emotional exhaustion, ethical ambiguity, and quiet forms of resistance that result. Theme 3, Relational Context and the Ethics of Euthanasia Decision Making, examines how the presence or absence of a responsible human counterpart—be it an owner or informal guardian—shapes ethical reasoning, decision-making processes, and the distribution of emotional responsibility. Theme 4, The Unequal Burden of Street Dog Euthanasia, focuses on the moral solitude that veterinarians experience when euthanizing street dogs, underscoring how the absence of relational support transforms these decisions into ethically fraught and emotionally isolating acts. Theme 5, Bearing Witness—Emotional Labor and Relational Strain in End-of-Life Encounters, reveals how veterinarians engage with and absorb the grief of those who care for dogs—whether owners or street dog guardians—highlighting the empathic labor involved in mediating human–animal loss. Finally, Theme 6, Making Time for Dignity of Death for Dogs—Veterinarians’ Expectations for Euthanasia Practice, brings forward practitioners’ visions for more humane, dignified, and reflective euthanasia practices, including small rituals, pauses, and affective gestures that assert care even in the face of institutional limitations. Together, these themes offer a complex, nuanced, and relationally situated account of veterinary euthanasia as practiced and experienced in the emotionally and ethically charged landscape of Istanbul.

- Theme 1: Shouldering the Burden—Euthanasia as a Moral Responsibility in Veterinary Practice

The first theme arose out of the personal and professional investment in performing euthanasia, as articulated in the participants’ accounts. These accounts provide a glimpse into the ways in which veterinarians internalize, legitimate, and emotionally appropriate the work of ending an animal’s life—alone, and always with consequence. For most, euthanasia is not so much a medical action; instead, it is a highly affective and morally significant action that pervades their whole relation to veterinary medicine.

For coding of these accounts, NVivo 12 Plus was employed in a two-step process. In the initial cycle, line-by-line in vivo and descriptive coding were carried out to maintain affectively rich phrases such as “it stays with me,” “the hardest part of my job,” or “I carry their death with me.” These initial codes revolved around veterinarians’ accounts of emotional intensities, ethical duty, and building professional identity. In the second cycle, pattern coding was performed to group repeated themes of normalization of euthanasia, emotional exhaustion, and setting up moral boundaries into the wider conceptual categories of “emotional imprint,” “protective ethics,” and “professional dissonance.”

Most of the respondents characterized euthanasia as an inevitable aspect of the work but not one that they ever reconciled themselves to. Many discussed it as a sort of ethical burden that had become second nature but was still troubling on an emotional level. One veterinarian explained the additive development of emotional baggage:

“Sometimes you forget the dogs, but sometimes you don’t. It’s not just death—it’s that you made the decision. That’s what stays with me”.

(VET12, female, self-employed, 11 years of experience)

Other veterinarians stressed that their euthanasia threshold altered over time, frequently becoming more emotionally guarded than calloused. One interviewee said: “I thought I’d got used to it. But I haven’t. In fact, I’ve got to be more careful. I take longer, I ask more. That weight hasn’t gone away—it’s just less obvious” (VET06, male, municipal shelter, 9 years of experience).

Most felt a strong moral obligation, particularly when euthanasia was requested for dubious reasons such as convenience, behavioral issues, or cost. They would then usually decline the request and take responsibility themselves for the animal’s care. In the words of one vet:

“I am not able to put down a dog simply because the owner no longer wants it. If she is curable or the animal is just old, I take them in and complete their treatment at my clinic”.

(VET12, female, employed, 11 years of experience)

These accounts reflect what has been widely recognized in the literature as moral distress: a psychological state that arises when veterinarians are compelled to act against their ethical convictions due to institutional or contextual constraints [27,28,29,30,69]. Some practitioners have described various coping mechanisms for navigating this ethical tension, including internal rationalization, passive resistance, or subtle forms of refusal (e.g., delaying procedures, proposing alternative treatments, or quietly withholding immediate compliance).

For many veterinarians, the process of euthanasia for companion dogs was more apt to include collaborative decision making, with veterinarians consulting with the local caregivers or guardians of the dog, considering palliative approaches, and monitoring their emotional preparedness closely. Such cases were more likely to be categorized as “relational euthanasia” and “painstaking pacing.” As one vet described: “Sometimes the dog is ready to go. But the owner is not. Then, I slow everything down—explain more, sit with them, give them time. I think that helps both the dog and the person who sees the dog as family member” (VET04, female, self-employed, 14.4 years of experience).

In contrast, the euthanasia of street dogs was consistently portrayed as a solitary decision, carried out in the absence of relational dialogue or caregiver involvement. These situations were marked by a profound sense of emotional solitude, often permeated by moral anguish, internal conflict, and lingering guilt. The distinction between euthanasia as a shared, deliberative process and as an isolated professional obligation became a central framework through which veterinarians made sense of its ethical and emotional weight. As one veterinarian articulated:

“When you’re on your own, you’re the only one who’s going to be making decisions. That sort of responsibility. It isn’t the same. It’s more… more responsibility… because there is no one else taking responsibility”.

(VET10, female, working, 3.5 years of experience)

This theme also highlights the contention that euthanasia is greater than a medical endpoint; rather, it is a battleground of ethical nuance where care, control, bereavement, and responsibility converge. It also illustrates how vets—far from being callous—operate in this context with tremendous sensitivity, frequently at their personal expense. Not surprisingly, a participant encapsulated this idea as follows:

“Euthanasia isn’t something you get used to. You just carry it differently… Every time, a different dog, different situation… I remember every euthanasia I had to make… All those last moments, I keep thinking…”.

(VET21, female, employed, 4 years of experience)

- Theme 2: Ethical Strain and the Emotional Costs of Adverse Euthanasia

This theme asks how the moral and emotional costs of performing euthanasia—especially when motivated by structural demands instead of clinical requirements—affects veterinarians’ emerging professional identity. Instead of perceiving such difficulties as being at the margins or the exception, most of the participants framed them as definitional for practicing veterinary medicine in morally difficult and institutionally bounded contexts. The emotional grooves of uncertainty, guilt, remorse, and isolation that became automatic were less residues of the procedure than formative encounters that intensified the practitioners’ moral awareness and marked out the boundaries of what they could—and what they could not—accept as care.

One vet recalled a particularly vivid moment that stuck with her:

“It wasn’t out of mercy. That dog did have a chance, but there was no room, there was no one to take care of him, and I was told to do it. That’s an euthanasia that I will never forget… I don’t like to do euthanasia after that dog anymore”.

(VET15, male, employed, 15.8 years of experience)

These ideas are corroborated by empirical accounts of moral distress in veterinary practice. As pointed out by Moses et al. [10] and Cooney et al. [13], moral distress typically occurs when veterinarians cannot act on their ethical discernment, especially when institutional or structural mandates take over clinical thinking. This type of negative euthanasia, where animals are put down due to reasons such as insufficient shelter space, limited funds, or municipal ordinances, leads to burnout and emotional exhaustion [27,28,29,30,69,91].

In the present study, NVivo 12 Plus was employed to code the qualitative data through a two-stage coding process. Line-by-line descriptive and in vivo coding within the first cycle was conducted to accurately capture the participants’ statements and highlight emotionally significant or ethically pertinent phrases such as “routine killing,” “forced to decide within minutes,” and “sacredness of the act.” As a result, codes including “institutional pressure,” “adverse euthanasia,” “ethical regret,” and “resistance efforts” were instantiated.

The second stage of analysis employed pattern coding to aggregate these descriptive codes into higher-order conceptual categories. Bureaucratic urgency, underfunded shelters, or sluggish deliberation codes were condensed into more general concepts of “systemic acceleration,” “ethical rupture,” and “resistance and moral agency.” Query functions and memo-linking properties served to map where sites of ethical unease coincided with structural constraints or lack of human buffering, including multiple-layered interdependencies between care, control, and constraints.

The veterinarians were quick to characterize the time frame within which euthanasia decisions needed to be made as a cause of ethical tension:

“We’re not really given the time to think long—some days, we have to know in a matter of minutes whether or not a dog is alive or dead”.

(VET08, male, self-employed, 11.9 years of experience)

Others labored to make ethical space within tight systems. One respondent described her experience as follows: “I worked with the shelter manager to institute a 48 h hold time rule before euthanasia. I do not always follow the rule, but it saved a couple of dogs” (VET20, female, self-employed, 18.9 years of experience).

The interviewees also referred to acts of individual resistance, such as stalling procedures, paying for treatment, or networking with rescue groups, as an act of reclaiming moral agency: “Sometimes I test the limits—I hold up on the decision, call in buddy rescues, or even write out of my own pocket. I can’t always make it happen, but I don’t want to give up so easily” (VET14, male, employed, 14.1 years of experience).

These fleeting instances of resistance capture how veterinarians fight against and sometimes subvert the routinization of death. They also carry a great emotional price tag. One interview participant described thus:

“No animal should be put to sleep because there is no money, no one stands for her, no animal should be killed if there is any possible cure. Euthanasia should not be chosen due to the quotas or full cages. My wish is to maintain my compassion—to never lose the mercy for the animals”.

(VET04, female, self-employed, 14.4 years of experience)

Ultimately, this theme underscores that euthanasia in such contexts is not just a clinical act—it is crucible for ethical deliberation and emotional labor. Euthanasia of street dogs becomes a practice shaped as much by infrastructural scarcity as by individual moral resolve. As one veterinarian poignantly observed: “Euthanasia becomes less of a decision and more of the state of the world we’re living and working in. That is what scares me” (VET25, male, self-employed, 9.2 years of experience).

- Theme 3: Relational Context and the Ethics of Euthanasia Decision Making

The third theme explores the layered clinical, ethical, and contextual considerations that shape veterinarians’ decision-making processes regarding euthanasia. The participants emphasized that while clinical indicators—such as pain severity, prognosis, disease progression, or lack of mobility—are foundational, they are never sufficient by themselves. Instead, each decision unfolds within a complex terrain shaped by the presence or absence of a human interlocutor, available care infrastructure, financial possibilities, and emotional implications.

To clarify the terminology in this article, two key terms are employed: the term owner refers to individuals who are legally and financially responsible for a dog, often maintaining a long-standing, emotionally intimate relationship. The term guardian, in contrast, refers to individuals who care voluntarily for street dogs (e.g., street feeders, volunteers, or neighbors). Although many guardians exhibit deep emotional bonds with animals, their relationships lack legal standing, institutional authority, or often the financial capacity to make end-of-life decisions.

Many veterinarians report that the emotional and ethical burden of the decision becomes more manageable when an owner is present. The shared nature of the process—including discussing the dog’s condition, deliberating over options, and making the final choice—offers both practical support and moral reassurance. “When the owner is there, even if it is a hard conversation, it helps to share the heavy feeling…” one vet noted. “Those dogs are lucky if they have someone…. If there’s someone to witness … and say goodbye” (VET09, male, self-employed, 6.6 years of experience).

In contrast, street dogs often arrive with no clearly accountable or stable human companion. Sometimes, guardians provide transport, temporary shelter, or food but are unable or unwilling to take long-term responsibility. This leaves veterinarians in an ethically precarious position. “Sometimes it’s a volunteer, sometimes a neighbor who feeds the dog,” one vet explained. “They bring the dog… I know they do their best to help an injured or sick street dog… ask for our help but disappear after that. People leave you with the final decision… After that, it is our responsibility… The entire burden is ours…” (VET16, female, self-employed, 15.6 years of experience).

Another vet elaborated: “I had a case where the dog had chances to live… it was not entirely impossible… but he would have to stay and maybe take years of treatment… but the guardian made it very clear that she could not afford the treatment. I continued the dog’s treatment for six more months… the dog was depressed, closed himself… then I had to do what the system, bigger institutions or local caregivers could not do… The dog needed relief… So I had to euthanize the dog… It was terrible. To this day, I still think about that dog…” (VET14, male, employed, 14.1 years of experience).

In these cases, euthanasia often becomes an ethically fraught and emotionally isolating decision that is not buffered by shared deliberation or institutional support. The absence of a responsible party or viable care alternatives leaves practitioners alone with decisions that feel less like clinical interventions and more like moral reckonings. “It’s lonelier,” said one municipal shelter veterinarian. “You hold the syringe, and there’s no one to ask—no one who will carry this with you” (VET12, female, employed, 7.9 years of experience).

To capture and analyze these layered accounts, an iterative, multi-cycle coding process was performed. In the first cycle, descriptive analysis and NVivo 12 Plus coding were employed, maintaining the veterinarians’ exact wording and preserving emotionally significant expressions such as “stuck between options” or “left alone with the decision.” These codes were grouped around themes such as clinical uncertainty, caregiver absence, economic limitations, and compassion fatigue. In the second cycle, pattern coding and focused coding were applied to refine and synthesize these clusters. Codes referencing financial hardship, lack of referral options, or institutional absence were subsumed under thematic categories such as “systemic constraints,” “ethical improvisation,” and “relational solitude.” Meanwhile, codes around co-decision making with owners were grouped under “shared responsibility,” while accounts of unassisted decision making were labeled “ethical isolation.” NVivo’s matrix coding, query, and memo-linking features facilitated cross-referencing of prognosis, dog status (owned vs. unowned), and decision burden factors, allowing for analysis of how different relational configurations shaped the participants’ ethical deliberations.

Empirical evidence gathered under this theme challenges any assumption that euthanasia is a neutral clinical endpoint. Instead, participants described canine euthanasia as a profoundly context-dependent and relational act, which is especially shaped by the presence or absence of a human guardian in the decision. When the owner or human guardian of the dog was involved, the process felt shared; even if painful. When guardians were absent or only temporarily engaged, not taking responsibility for the entire process of medical treatment, veterinarians were left to navigate the emotional and ethical weight alone. This was particularly salient in Istanbul’s urban landscape, where municipal services for street dogs are fragmented, medical infrastructure and services are only limited, and veterinary clinics—especially private ones—are often left to cover for institutional failures.

Ultimately, this theme demonstrates that ethical decision making in euthanasia is not a matter of predetermined protocol but of situated judgment—formed through tripartite relationships (dog–guardian/owner–veterinarian), constrained by institutional and financial challenges, and often guided by the veterinarian’s ethical compass under conditions of uncertainty, stress, and emotional isolation.

- Theme 4: The Unequal Burden of Street Dog Euthanasia

Veterinarians consistently described euthanizing street dogs—that is, dogs without legal owners or stable guardians—as being deeply different from companion animal cases. In these cases, ethical and emotional burdens are magnified due to the absence of relational support or the human owner taking responsibility. The act of euthanasia becomes a solitary moral space in which veterinarians feel that they bear full responsibility.

“When there’s no guardian, it’s like I become the only moral presence in the room. That loneliness is heavy”.

(VET13, male, self-employed, 17.4 years of experience)

During the first NVivo coding cycle, emotionally intense phrases—such as “alone in the decision,” “nobody to ask,” and “no one to speak for them”—were tagged frequently. These in vivo codes formed the basis of nodes including “relational absence” and “ethical ambiguity.” In the second cycle, pattern coding and focused coding linked these nodes with broader themes including “systemic constraints” and “asymmetrical responsibility.” NVivo’s query tools revealed that themes of loneliness, grief, and isolation were significantly more prevalent in transcripts about street dog cases than when guardians were present.

Veterinarians expressed acute discomfort over ending lives without shared emotional acknowledgment: “The hardest part is not knowing their story. You end a life that had no one to speak for it” (VET24, female, employed, 20.3 years of experience).

With no owner present, vets described acting as judge, witness, and executor in one: “I become judge, witness and executioner all at once. It’s not a role anyone should bear alone” (VET06, female, employed, 14.2 years of experience).

Participants noted that these decisions do not just weigh on them momentarily—they accumulate over time, leaving emotional residue. In contrast, when a legal owner is involved, euthanasia becomes a shared decision. The presence of an owner not only distributes grief and responsibility but also offers a relational framework in which death is contextualized, justified, and emotionally contained.

There were, however, some attempts at resisting the imposed isolation. One veterinarian introduced a 48 h pause rule before euthanizing street dogs, providing time for reflection. Though inconsistently applied, it was described as a potential model of more humane decision making (VET20, female, self-employed, 18.9 years of experience). Another vet recounted an experience of delaying euthanasia by taking a paralyzed dog home and sharing the story online, ultimately facilitating its adoption (VET07, male, self-employed, 19.1 years of experience).

The experiences shared by veterinarians in this study echo what much of the veterinary ethics literature has already begun to uncover: euthanasia—especially when performed without the presence of a guardian or owner—can be one of the most morally and emotionally taxing parts of clinical practice. When a dog has no legal owner or even an informal caregiver to speak for them, veterinarians often find themselves alone in making life-ending decisions. These moments, as many participants described, carry a particular weight.

Research supports these lived realities. In a North American survey, Moses et al. [10] found that when veterinarians feel they cannot act in line with what they believe is ethically right—often due to financial limits or a lack of institutional support—they experience deep moral tension. The burden becomes even heavier when there is no one to share the responsibility of the decision, no one to confirm that it is time, no one to grieve alongside. In such moments, the veterinarian is not just performing a procedure—they are carrying the full emotional and ethical weight of the decision.

Several participants pointed to this feeling of ethical solitude, especially when treating street dogs were brought to the clinic by volunteers or municipal officers. As one veterinarian put it: “They bring the dog in, and then they’re gone. You’re left with the dog, the prognosis, and no clear next step. And then it’s on you—just you” (VET08, male, self-employed, 11.9 years of experience). In the absence of a shared decision-making process, many vets described feeling like they were being asked to act as both doctor and moral guardian. Christiansen et al. [96] have noted how important collaboration is in end-of-life decisions; not only for honoring the animal’s life and history, but for helping the veterinarian to bear the emotional burden of that decision. When the relational structure is absent, the decision becomes harder, lonelier and, in many cases, more ethically troubling.

Ultimately, what this theme reveals is that euthanasia in these cases is not just a clinical procedure: it is a deeply human moment shaped by uncertainty, loss, and systemic failure. In a city such as Istanbul, where formal infrastructures for the end-of-life care of street dogs are weak or absent, private practitioners often find themselves making the best decisions they can with what little support they have. While they may act with compassion and skill, they do so within systems that rarely acknowledge the emotional cost of this work.

However, the transferability of these findings should be approached with caution. Istanbul’s legal framework—particularly the recent 2024 amendments in the Animal Protection Law [50] and the dense urban street dog context—create a distinctive institutional environment. Nonetheless, the ethical challenges observed—that is, navigating euthanasia decisions under conditions of structural fragmentation, weak professional guidance, and heightened public scrutiny—may also arise in other large metropolitan contexts where municipal veterinary services manage free-roaming dog populations under comparable conditions. Comparative research on urban animal governance has highlighted that such municipalities frequently encounter similar challenges, including fragmented institutional responsibilities, inconsistent regulatory frameworks, and limited professional guidelines for veterinary decision making [33,97,98]. These shared structural constraints suggest that, while Istanbul presents a unique case, the ethical tensions identified in this study may resonate across diverse global contexts facing similar governance and resource pressures [99,100,101,102,103].

- Theme 5: Bearing Witness—Emotional Labor and Relational Strain in End-of-Life Encounters

The veterinarians in this study emphasized that euthanasia is never just a clinical act: it is an encounter steeped in emotion, shaped not only by the dog’s condition but also by the reactions of the owners, guardians, or informal caregivers who cared for it. Managing these reactions emerged as one of the most emotionally demanding parts of the process. Grief, they explained, is contagious—it seeps into the room and often lingers long after the procedure is over.

The ways in which veterinarians responded to that grief varied, depending on who was present and how the relationship between the human and the animal was understood. With pet owners—that is, those with long-standing legal and emotional bonds to the dog—veterinarians described slipping into what felt like a familiar, if difficult, role. They knew how to speak gently, when to pause, and when to give space. One vet put it plainly: “I always speak calmly to pet owners. They’re not just clients; they’re family to that dog. Even when I’m exhausted, I try to meet them where they are” (VET18, female, employed, 4.4 years of experience).

However, the emotional stakes shifted most visibly in relation to street dogs, as animals who lack identifiable owners and, often, any stable or enduring caregiver. In Istanbul, their survival depends on diffuse and precarious networks of care: a neighbor who leaves out food, a shopkeeper who builds a makeshift shelter, or an activist who intervenes against harm. These everyday practices of feeding, sheltering, and protecting do not amount to formal guardianship, yet they create a fragile web of recognition and responsibility. When such dogs arrive at the clinic, veterinarians confront not only the animal’s medical condition but also the uneven and often ephemeral traces of human care that accompany them. The absence of a singular accountable guardian amplifies the ethical weight of euthanasia, transforming it into a decision made in the shadow of communal—but ultimately fragmented—caregiving.

Informal caregivers, local animal protection volunteers, neighbors, or concerned passersby often arrive at the clinic in visible distress, sometimes having been the only person who had cared for the dog. Their mourning, as veterinarians noted, was quieter, often overlooked, and occasionally laced with guilt or helplessness. “Pet owners are consoled by family and rituals,” said one vet. “Those who take care of street dogs, ordinary animal lovers usually mourn alone. And I feel that. I try to hold space for their grief in a different way…. Sometimes they get bitter, they talk bitter and regretfully… and blame the veterinarian for what has happened” (VET09, male, employed, 6.6 years of experience).

Another vet reflected: “If the dying animal is street dog and the caregiver breaks down, I try to stay composed, but sometimes their pain becomes mine too. A street dog has a small chance of surviving, and I feel that I took away that chance” (VET21, male, employed, 6.5 years of experience). Many participants expressed a particular tenderness toward these moments, recognizing the emotional courage it takes to care for a street dog and then to witness its death.

In the first cycle, descriptive coding helped to capture the participants’ exact words and emotionally loaded expressions, including phrases like “held back my tears,” “mirrored their sadness,” or “slowed my tone.” These were grouped under initial codes such as “emotional containment,” “mourning response,” and “relational empathy.” In the second cycle, pattern coding was performed to draw connections between types of caregivers and the emotional labor involved. Codes related to tone modulation, pacing, bodily posture, and intensity of perceived grief were brought together under broader themes such as “affective attunement,” “shared sorrow,” and “compassion strain.”

What stood out in many of these accounts was how deeply the veterinarians felt drawn into the emotions of others; not because they were unprofessional, but because being emotionally present felt like part of the job. Dog euthanasia—particularly in the case of a street dog—was not always something they were trained for, nor was it explicitly valued within institutional frameworks. However, how a veterinarian responds to those people who are concerned about the life and death of a dog significantly matters. One veterinarian described it this way: “When someone is crying for a street dog, I lower my voice. I sympathize, I try to consol the person… I move slower, I give that person time and space. That’s the least I can do for them—and for the dog” (VET11, male, self-employed, 1.9 years of experience).

This kind of emotional labor—meeting another’s grief while managing your own—has been recognized in the veterinary literature as a source of both meaning and vulnerability. Studies have shown that veterinarians who perform euthanasia regularly are at increased risk of compassion fatigue and moral stress, particularly in contexts where emotional support systems are lacking [10,12]. As this theme suggests, such risks are intensified in moments when veterinarians become the sole witness to both the dog’s passing and the caregiver’s mourning.

In the quiet after the procedure—once the client has left or the street dog’s caregiver walks away—veterinarians are often left alone with what one called “the emotional residue.” It is not just about ending a life; it is about holding the emotional weight of that ending for someone else, while also carrying your own.

- Theme 6: Making Time for Dignity of Death for Dogs—Veterinarians’ Expectations for Euthanasia Practice

This theme focuses on how veterinarians envision an alternative type of end-of-life care: one that makes room for empathy, reflection, and dignity, even in clinical systems that do not always allow for such things. Many participants spoke not about what euthanasia is, but instead how it could be, if time and support permitted.

The veterinarians commonly expressed their professional aspirations in muted, individualized terms. One stated: “I want every euthanasia to be one that I could explain to the dog, if they could hear” (VET16, female, self-employed, 12 years of experience). In another brief silence, she stated “It doesn’t have to be some big thing—a blanket, a look in the eye, a minute to breathe before the injection. Killing of a dog should not be rushed. That matters” (VET15, male, self-employed, 15.8 years of experience).

These small details—soft blankets, a sympathetic tone of voice, somewhere to sit afterwards—were ways of respecting the solemnity of the moment. One vet remembered setting up a “grief corner” in their clinic, a small area with tissues and dim lighting where people could sit with their grief (VET01, male, self-employed, 4.5 years of experience). These were not decisions of protocol but of personal ethics: acts of care that make euthanasia feel like more than a procedure.

Others discussed what was yet to be had. A number of veterinarians discussed the necessity of hospice care, particularly for street dogs with no one to sit with them. “We need somewhere they can go when it’s not quite time yet… They shouldn’t need to be euthanized simply because there isn’t space to lie down,” one veterinarian commented (VET19, male, self-employed, 18.2 years of experience). Another remarked, “I have a dream about a hospice place, just a week or two, where they can be seen, and known, and maybe even recover. Or at least, get out on their own terms” (VET06, female, employed, 14.2 years of experience).

These wishes were made clear through the NVivo 12 Plus analysis. In the initial coding cycle, the phrases “blanket for goodbye,” “moment of silence,” and “this should mean something” emerged. These in vivo codes were sorted into themes of ritual care, slowness, and respect in death. In the second cycle, more general categories of end-of-life ethics and future care imaginaries assisted in linking these minor acts to a general moral position of undoing emotional detachment.

What these stories disclosed was not naïve idealism but a somewhat less manifested determination not to break the emotional continuity of their work, despite fatigue or pressure. “I’ve had bad days,” one vet explained, “but I’ll stop. Even for thirty seconds. I hear the last breath of the dog. And put my hand on the head of the dog. Pet the dog, one last time. That’s what I’d want if it were for me” (VET26, female, employed, 15.4 years of experience).

This theme reveals that, for most veterinarians, euthanasia is not merely about ending the suffering of the dog, it is also about *how* that ending occurs. It is about making space for what matters, the moment of death, a dog’s passing, particularly when there is little remaining that the veterinarian can offer. These modest acts—whether a touch, a hesitation, or a refusal to hurry—convey the emotional weight of ending an animal’s life with dignity; particularly in the case of a street dog, where the veterinarian is solely responsible. For many veterinarians, the moments during and after a street dog is euthanized are reminders that, even at life’s end, dignity is something that can still be offered.

## 4. Discussion

Euthanasia was not portrayed by veterinarians in this research as part of ordinary clinical practice. They discussed euthanasia as something that shapes, haunts, and quietly reshapes them throughout the years. Their evidence reveals a practice shrouded in layers of emotional, ethical, and institutional richness, where even mundane procedures are infused with meaning and meaningfulness. By listening to these testimonies, one may observe how euthanasia—especially when defined within the fragile animal welfare framework of Istanbul—cannot be reduced to the terminal point of treatment. Rather, it is much more intricate and multifaceted, forming a space where ethical principles, relational presence, and the boundaries of care are constantly negotiated.

While this article uses the term euthanasia to describe the veterinary practice of ending a dog’s life, it is important to acknowledge that the applicability of this concept to non-human animals is contested. Several scholars, as well as some veterinarians interviewed as part this study, argue that the term euthanasia—rooted in notions of voluntary, consented “good death” in human medicine—becomes ethically problematic when extended to animals who cannot express informed consent or indicate a personal will regarding their dying. From this perspective, the term risks obscuring the structural and relational asymmetries that underpin decisions about ending an animal’s life, particularly in contexts involving street dogs with no formal guardianship. Nevertheless, the article adopts the term euthanasia strategically and analytically to situate the findings within the existing literature on veterinary ethics and to engage with debates on end-of-life decision making in both companion animal and street dog contexts. The aim is not to endorse a normative position on whether the practice constitutes a “good death” but, rather, to foreground the nuances, moral ambiguities, and emotional burdens that veterinarians experience when navigating these decisions.

Several of the participants stressed that they never got “used” to euthanasia, regardless of how frequently they had done it. Taking a being’s life, particularly beyond obvious clinical necessity or in the midst of relational scarcities, was found to be ethically burdensome and emotionally affective. Such accounts have been reported by Moses et al. [10], who conceptualized moral distress in veterinary professionals as a circumstance that arises when clinicians cannot act in response to their ethical opinions due to external constraints. Whereas the idea of the “caring–killing paradox” [1] has been a common point of reference in the literature, what unfolded in this study was not only related to the inner contradiction of killing in the name of care but also the emotional aftermath of performing it repeatedly and frequently without the necessary emotional support, open and understanding deliberation and shared decision making, or even consultation with other colleagues and animal care professionals that would otherwise facilitate the emotional processing of the veterinarians.

Responsibility here is not an abstract construct; it is embedded in the flesh. For example, when a dog departed, the presence of a caretaker—regardless of whether they were a long-time owner or a local volunteer—was reported to soften the emotional burden. There was someone to advocate for the creature, someone to mourn with, someone to acknowledge the time. In these moments, the veterinarians spoke of themselves as partners in a mutual ethical endeavor, although this was still not easy. In contrast, when the dog was a street dog—either alone or brought in by a concerned but uninvolved stranger—that moment of mutual moral presence collapsed. Respondents kept returning to the words “being the only one” or “being alone in the room.” This ethical isolation—being both the decision maker and the one who had to grieve—made a distinct impression; one that Montoya et al. [75,76] have reported as being particularly challenging when relational context is absent.

Some veterinarians spoke of having to euthanize animals that would have survived if there had been space, necessary financial sources, or extended care for the dogs. These were the things that stuck with them—not that the animal was injured, but that they knew it was a collapse of structure and not a clinical decision. These recalls align with Kipperman et al. [12], who defined adverse euthanasia as ending life not out of compassion but because there were no other viable alternatives. For others, they were presented as betrayals of the very ethics of care that had led them into veterinary practice. Yet, even in these ethically compromised scenarios, there were moments of quiet resistance as well. The participants talked of putting off procedures, making do with ad hoc hospice spaces, raising money for care, or simply waiting—just to give the dog a little more time, and themselves a little more moral space to breathe. These small acts of resistance resonate with the study of Bartram et al. [29] on the exercise of individual conscience in the face of institutional normalization of ethically disconcerting practices.

One particularly striking example (Lines 812–813) illustrates how veterinarians negotiate the boundary between personal and professional ethics. The veterinarian framed the act of “saving” not only through clinical judgment but also through their personal moral conviction, comprising an instance where psychological egoism—that is, the pursuit of what aligns with one’s own moral sense of self—appears to overlap with a professional commitment to an ethic of care. These moments suggest that euthanasia decisions are not only governed by professional guidelines but are also deeply shaped by veterinarians’ self-understanding of moral agency and responsibility. While a full philosophical exploration of this tension lies beyond the scope of the present paper, it is important to acknowledge that personal ethical reasoning often affects and sometimes complicates the enactment of professional norms in veterinary practice. This interplay underscores the richness—and the messiness—of moral reasoning in everyday euthanasia work.

These findings challenge the assumption that euthanasia decisions in veterinary ethics can be universally governed by normative principles such as “minimizing suffering.” While this principle remains foundational, it fails to account for the complex, uneven conditions under which veterinarians practice; particularly in cases involving street dogs, where institutional support is lacking, relational responsibility is absent, and the veterinarian is left to carry the ethical weight alone. In such cases, moral reasoning becomes situational, being contingent on context rather than the application of abstract rules. This supports Linder et al.’s [104] argument that veterinary ethics requires a model of practical judgment rooted in the specifics of each case, rather than rigid adherence to decontextualized principles. Similarly, Kipperman et al. [12] documented the ethical tensions that arise when clinical limitations and client expectations constrain the actions of veterinarians; yet, the moral challenges identified here go further by showing how ethical agency is reconfigured in the absence of any client or guardian. As Linder et al. [104] emphasized, incorporating empirical realities into normative frameworks is essential for ethics to remain responsive and credible. This study contributes to that goal by foregrounding how everyday moral reasoning unfolds in constrained and asymmetric contexts; particularly those involving the euthanasia of street dogs, where the logic of care, responsibility, and control is radically re-defined.

These findings also resonate with what Arluke et al. [105] conceptualized as the sociozoological scale, which classifies animals based on human cultural categories of worthiness, belonging, and legitimacy. In the context of Istanbul, the same dog may occupy shifting positions on this scale—at one moment a “street dog,” at another a “community companion” and, when adopted, a “family pet.” The ethical and emotional work of veterinarians often reflects these shifting statuses. As the participants emphasized, the decision to euthanize was rarely reducible to a clinical prognosis alone; it was also shaped by whether the animal was socially recognized as a subject of care and relational responsibility. While the present study does not take the sociozoological scale as its central analytical framework, situating its findings in this context highlights how deeply entangled veterinary ethics is with broader cultural hierarchies of animals’ value and belonging.

Emotional labor performed in end-of-life interactions was also foregrounded in the participants’ experiences. Whether whispering to a mourning owner or speaking to a sobbing street dog caretaker, vets not only perform the procedure but also soak up and facilitate the other person’s sorrow. One vet spoke of empathetic understanding of the caretaker’s grief, another timing the conversation to permit silence. These were never necessarily formally taught in school or given a particular name in formal ethics codes but were at the forefront of how the participants thought about “good practice.” A veterinarian’s experience of conducting euthanasia is frequently more a matter of their attention to the relational and emotional aspects of death than technical adherence [2,17]. Even so, this attention frequently comes at their personal expense. The interviewed veterinarians were emotionally exhausted, often alone in their grief, and rarely afforded institutional recognition for the emotional toll they had paid. Burnout was mentioned by a few; others described episodes of detachment or emotional depletion. These results resonate with recent discussions in the broader literature on compassion fatigue [10,13], affirming the imperative to rethink what kinds of acknowledgment and support veterinarians need—not merely as professionals but as human beings who are constantly in close proximity to loss.

Significantly, the interviews also revealed a key empirical finding: gender did not appear to be a determining factor in how the veterinarians experienced or responded to the ethical challenges of euthanasia. Both male and female participants articulated similar emotional and moral struggles. However, it is important to note that some gendered dimensions of experience emerged inductively from participants’ narratives, rather than being derived from a pre-defined analytical focus on gender. This is consistent with the study’s reflexive thematic analysis approach [70,71,72,73], which prioritizes allowing patterns to emerge organically from participants’ accounts rather than imposing pre-established categorical frameworks. Although several woman veterinarians described experiencing heightened emotional strain in relation to euthanasia, compared to some of their male colleagues, these insights are presented here as contextual themes reflecting the participants’ own meaning-making processes rather than as systematically tested or statistically substantiated findings. In contrast, years of professional experience—particularly when coupled with independent practice—did appear to make a notable difference. Veterinarians who had transitioned into self-employment often described a greater sense of ethical autonomy and professional confidence. This accumulated experience seemed to function as an emotional and moral resource, enabling them to navigate both the procedural and affective dimensions of euthanasia with more clarity and less internal conflict. These veterinarians spoke of clearer boundaries, more reflective practices, and a deeper attunement to their own ethical thresholds. In this context, experience did not diminish the moral complexity of euthanasia but allowed practitioners to carry it with greater steadiness.

These findings deepen and expand upon a growing body of empirical work in veterinary ethics that highlights the emotional strain and moral complexity embedded in end-of-life decision making. Hartnack et al. [65] have emphasized the role conflicts that veterinarians often face when their caregiving responsibilities are constrained by institutional limitations, especially in the context of euthanasia. Similarly, Hannah et al. [66] underlined the importance of emotional regulation not as a peripheral skill but as a central, often overlooked component of veterinary professionalism. Quain and Quain et al.’s [3,4] calls for a form of veterinary ethics that is sensitive to moral imagination and context echoes the improvisational acts of care, hesitation, and resistance discussed by participants in this study. There is a growing shift in the field away from frameworks based solely on abstract ethical principles, instead moving toward approaches that are responsive to relational complexity and real-world ambiguities [5]. It is important to note that moral obligations in veterinary practice arise not only from fixed duties but also from the specific relationships, tensions, and circumstances that practitioners encounter. This study contributes to that conversation by offering a situated account of how moral reasoning unfolds in ethically dense and relationally thin situations—such as street dog euthanasia—in the context of which institutional backing is minimal and veterinarians often carry the burden alone.

The most compelling aspect in most of these narratives, however, was not despair but rather a vision—tenuous, often whispered, but abiding—of a more ethical mode of practice. Some of the vets explained slowing it down: turning down the light, spreading out the blanket, providing a moment’s hesitation before the injection. These small rituals were not administrative requirements; instead, they were individual acts of morality, a means of preserving a sense of humanity in the face of institutional callousness. Yeates and Yeates et al. [8,9] have highlighted the necessity for a more reflective practice of euthanasia, one which reconsiders ethical principles and permits pause, emotional processing, and moral awareness. The individual in-depth interviews conducted in this study also helped the veterinarians to reflect once again on their own experiences, which are often overlooked by the other humans involved (e.g., the owner, the caregiver, the guardian, other employees at the clinic, or other colleagues). Others hoped for larger spaces where sick dogs might go—not to be killed, but to be kept, to be seen, and to be offered a chance, or at least a pause that did not feel quite so final. These visions were not always realizable, but they were not unrealizable either. They took form through years of being in this kind of work, seeing the gaps in the system and still being able to hold on to an ethic of care that felt whole.

What this research ultimately reveals is that euthanasia is not one discrete action but a relational and ethical process. It happens differently based on who is there, what resources are available, and whether there is physical and metaphorical space available for the practitioner to move in accordance with their conscience. This study also uncovered the coarse differences in the handling of death among street and companion dogs, and the ways in which those differences emotionally resonate throughout the lives of their caregivers.

Placing the testimony of veterinarians at center stage without smoothing over their contradictions or abstracting away the muddiness of ethical life, this study aids in the development of a broadening topography of empirical veterinary ethics that resists abstraction and instead listens to lived experience. In this context, not only should the moral burdens experienced by veterinarians be taken into consideration but also their ethical imagination, their ability to think creatively, and their will to act in the right way even when the fragmented professional guidelines, expectations by some dog owners, political atmosphere around them seems to be conspiring against it. In this way, ethics in veterinary medicine is not about rules or consequences; instead, it is increasingly about the unique, context-specific, and social and emotional relations between the animal; the human owner, guardian, or caregiver; and the veterinarian. The relational nature of pre- and post-euthanasia processes is also implicated in shaping veterinarians’ experiences of deciding on, performing, and coping with the emotional burden of euthanizing companions and street dogs, affecting their professional identity and senses of duty, care, and understanding of the dogs’ right to live and their dignity.

## 5. Conclusions

In parallel with Springer and Grimm’s call [106] to examine the context-specific responsibilities of veterinary professionals, this research demonstrated that euthanasia in veterinary practice is not a uniform or neutral act but, instead, one that acquires distinct ethical and emotional contours depending on the status of the animal and the relational context in which decisions are made. When performed in the presence of an owner, euthanasia is often experienced as a shared, if painful, process marked by dialogue, mutual acknowledgment, and a distributed sense of responsibility. In contrast, the euthanasia of street dogs was repeatedly described by participants as being more ethically ambiguous and emotionally burdensome. In such cases, veterinarians frequently find themselves acting as the sole decision makers and witnesses, with no relational counterpart to consult, deliberate with, or grieve alongside. These differences underscore the asymmetries that characterize end-of-life care for companion dogs versus street dogs, particularly in urban contexts where institutional support for street animals remains limited. The findings suggest that the emotional and moral significance of euthanasia is not determined solely by medical prognosis but by the degree to which the act is socially and ethically shared. By foregrounding these distinctions, this study contributes to a more nuanced understanding of the ethical landscape of veterinary euthanasia and calls attention to the often-overlooked challenges faced by practitioners who must navigate care, judgment, and loss in contexts of relational absence and institutional failures.

Contrasting Istanbul’s professional and institutional constraints with international frameworks—such as the AVMA [55,98] and WSAVA [58] Guidelines—underscores an important dimension of transferability. While cities with established, standardized euthanasia protocols likely experience different dynamics, insights from Istanbul’s context contribute significantly to broader global debates on the relational, emotional, and ethical complexities of veterinary euthanasia decision making.

## 6. Future Directions

This study opens up several avenues for further empirical research into euthanasia practices, particularly in contexts that lie outside the conventional veterinarian–client–animal triad. Future studies would benefit from expanding their analytical focus beyond veterinarians or institutional policy frameworks to consider the broader interpersonal, spatial, and affective dynamics of the clinic. Veterinary clinics are shared workplaces shaped by layered and often unequal relationships among veterinarians, technicians, caretakers, reception staff, janitors, students, and volunteers. Each of these actors engages with the euthanasia process differently—emotionally, ethically, and practically—and contributes to the atmosphere in which end-of-life decisions are made and enacted. Ethnographic research is especially well-suited to exploring how these interpersonal dynamics influence not only the distribution of emotional labor but also the moral sense-making surrounding the killing of animals in institutional care.

Further research is also needed to address the underexplored phenomenon of adverse euthanasia, such as cases in which the procedure fails, where suffering is prolonged, or where ethical discomfort persists long after the event. These moments, though difficult to witness and even harder to narrate, are ethically significant. They highlight the fragility of care in high-pressure or under-resourced settings and complicate the dominant framing of euthanasia as a clean or merciful act. Attention to such events would allow for a more nuanced understanding of how veterinarians and related staff navigate uncertainties, errors, and moral residues.

Moreover, the emotional experience of euthanasia of companion and street dogs is not uniform and is shaped by factors such as age, gender, and professional role. Younger or less experienced staff are often delegated the most emotionally and physically taxing tasks. Female veterinarians, in particular, are frequently expected to provide not only technical care but also emotional support, especially in the absence of formal owners or guardians, as is often the case with street dogs. These dynamics reflect broader gendered and hierarchical patterns within veterinary care and deserve closer attention in the context of empirical ethics. Future studies focused on these perspectives—foregrounding not only professional authority but also overlooked, informal, or background roles—can deepen our understanding of euthanasia as a socially distributed, ethically complex practice. By taking the emotional texture and relational structure of the spaces in which animals are put to death seriously, empirical veterinary ethics can move toward a more situated, inclusive, and critically engaged account of care at the end of life.

## Figures and Tables

**Table 1:** Professional characteristics of the 29 veterinarians who participated in this study, including gender, years of professional experience, and employment type (self-employed or employed in private clinics).

Participant ID	Gender	Years of Experience	Employment Type
VET1	F	12	Self-employed
VET2	M	15	Employed
VET3	F	8	Self-employed
VET4	M	6	Employed
VET5	F	14	Self-employed
VET6	M	10	Self-employed
VET7	F	7	Employed
VET8	M	22	Self-employed
VET9	F	5	Employed
VET10	M	18	Self-employed
VET11	F	11	Self-employed
VET12	M	9	Employed
VET13	F	13	Self-employed
VET14	F	4	Employed
VET15	M	16	Self-employed
VET16	F	20	Self-employed
VET17	M	6	Employed
VET18	F	7	Self-employed
VET19	M	25	Self-employed
VET20	F	5	Employed
VET21	M	12	Self-employed
VET22	F	9	Employed
VET23	M	8	Self-employed
VET24	F	10	Employed
VET25	M	14	Self-employed
VET26	F	6	Employed
VET27	M	21	Self-employed
VET28	F	7	Employed
VET29	M	11	Self-employed

## Data Availability

The data presented in this study are available on request from the corresponding author. (The data are not publicly available due to privacy or ethical restrictions).

## References

[1] Reeve C.L., Rogelberg S.G., Spitzmuller C., Digiacomo N. (2005). The Caring-Killing Paradox: Euthanasia-Related Strain Among Animal-Shelter Workers1. J. Appl. Soc. Psychol..

[2] Matte A.R., Khosa D.K., Coe J.B., Meehan M.P. (2019). Impacts of the Process and Decision-making around Companion Animal Euthanasia on Veterinary Wellbeing. Vet. Rec..

[3] Quain A. (2021). The Gift: Ethically Indicated Euthanasia in Companion Animal Practice. Vet. Sci..

[4] Quain A., Mullan S., Ward M.P. (2022). Low and No-Contact Euthanasia: Associated Ethical Challenges Experienced by Veterinary Team Members during the Early Months of the COVID-19 Pandemic. Animals.

[5] Rollin B.E. (2011). Euthanasia, Moral Stress, and Chronic Illness in Veterinary Medicine. Vet. Clin. N. Am. Small Anim. Pract..

[6] Kondrup S.V., Anhøj K.P., Rødsgaard-Rosenbeck C., Lund T.B., Nissen M.H., Sandøe P. (2016). Veterinarian’s Dilemma: A Study of How Danish Small Animal Practitioners Handle Financially Limited Clients. Vet. Rec..

[7] Bussolari C.J., Habarth J., Katz R., Phillips S., Carmack B., Packman W. (2018). The Euthanasia Decision-Making Process: A Qualitative Exploration of Bereaved Companion Animal Owners. Bereave. Care.

[8] Yeates J.W., Main D.C.J. (2011). Veterinary Opinions on Refusing Euthanasia: Justifications and Philosophical Frameworks. Vet. Rec..

[9] Yeates J. (2010). Ethical Aspects of Euthanasia of Owned Animals. Pract. Manag..

[10] Moses L., Malowney M.J., Wesley Boyd J. (2018). Ethical Conflict and Moral Distress in Veterinary Practice: A Survey of North American Veterinarians. J. Vet. Intern. Med..

[11] Kipperman B., Rollin B., Martin J. (2021). Veterinary Student Opinions Regarding Ethical Dilemmas Encountered by Veterinarians and the Benefits of Ethics Instruction. J. Vet. Med. Educ..

[12] Kipperman B., Morris P., Rollin B. (2018). Ethical Dilemmas Encountered by Small Animal Veterinarians: Characterisation, Responses, Consequences and Beliefs Regarding Euthanasia. Vet. Rec..

[13] Cooney K., Kipperman B. (2023). Ethical and Practical Considerations Associated with Companion Animal Euthanasia. Animals.

[14] Christiansen S.B., Kristensen A.T., Lassen J., Sandøe P. (2015). Veterinarians’ Role in Clients’ Decision-Making Regarding Seriously Ill Companion Animal Patients. Acta Vet. Scand..

[15] Knesl O., Hart B.L., Fine A.H., Cooper L., Patterson-Kane E., Houlihan K.E., Anthony R. (2017). Veterinarians and Humane Endings: When Is It the Right Time to Euthanize a Companion Animal?. Front. Vet. Sci..

[16] Kuře J., Kuře J. (2011). Good Death Within Its Historical Context and as a Contemporary Challenge: A Philosophical Clarification of the Concept of “Euthanasia”. Euthanasia—The “Good Death” Controversy in Humans and Animals.

[17] Bubeck M.J. (2023). Justifying Euthanasia: A Qualitative Study of Veterinarians’ Ethical Boundary Work of “Good” Killing. Animals.

[18] Morris P. (2012). Managing Pet Owners’ Guilt and Grief in Veterinary Euthanasia Encounters. J. Contemp. Ethnogr..

[19] Kogan L.R., Cooney K.A. (2023). Defining a “Good Death”: Exploring Veterinarians’ Perceptions of Companion Animal Euthanasia. Animals.

[20] Cohen S.P. (2007). Compassion Fatigue and the Veterinary Health Team. Vet. Clin. N. Am. Small Anim. Pract..

[21] Hutton V.E. (2019). Animal Euthanasia—Empathic Care or Empathic Distress?. Vet. Rec..

[22] Marchitelli B. (2019). An Objective Exploration of Euthanasia and Adverse Events. Vet. Clin. N. Am. Small Anim. Pract..

[23] Caffrey N., Mounchili A., McConkey S., Cockram M.S. (2011). Survey of Euthanasia Practices in Animal Shelters in Canada. Can. Vet. J..

[24] Nett R.J., Witte T.K., Holzbauer S.M., Elchos B.L., Campagnolo E.R., Musgrave K.J., Carter K.K., Kurkjian K.M., Vanicek C.F., O’Leary D.R. (2015). Risk Factors for Suicide, Attitudes toward Mental Illness, and Practice-Related Stressors among US Veterinarians. J. Am. Vet. Med. Assoc..

[25] Kahler S.C. (2015). Moral Stress the Top Trigger in Veterinarians’ Compassion Fatigue: Veterinary Social Worker Suggests Redefining Veterinarians’ Ethical Responsibility. J. Am. Vet. Med. Assoc..

[26] Deponti P.S., Jaguezeski A.M., Pulgatti D.H.V., Soares J.C.M., Cecim M.d.S. (2023). Veterinarian’s Perceptions of Animal Euthanasia and the Relation to Their Own Mental Health. Ciência Rural..

[27] Witte T.K., Spitzer E.G., Edwards N., Fowler K.A., Nett R.J. (2019). Suicides and Deaths of Undetermined Intent among Veterinary Professionals from 2003 through 2014. J. Am. Vet. Med. Assoc..

[28] Tran L., Crane M.F., Phillips J.K. (2014). The Distinct Role of Performing Euthanasia on Depression and Suicide in Veterinarians. J. Occup. Health Psychol..

[29] Bartram D.J., Baldwin D.S. (2010). Veterinary Surgeons and Suicide: A Structured Review of Possible Influences on Increased Risk. Vet. Rec..

[30] Tomasi S.E., Fechter-Leggett E.D., Edwards N.T., Reddish A.D., Crosby A.E., Nett R.J. (2019). Suicide among Veterinarians in the United States from 1979 through 2015. J. Am. Vet. Med. Assoc..

[31] Pohl R., Botscharow J., Böckelmann I., Thielmann B. (2022). Stress and Strain among Veterinarians: A Scoping Review. Ir. Vet. J..

[32] Dalum H.S., Tyssen R., Moum T., Thoresen M., Hem E. (2024). Euthanasia of Animals—Association with Veterinarians’ Suicidal Thoughts and Attitudes towards Assisted Dying in Humans: A Nationwide Cross-Sectional Survey (the NORVET Study). BMC Psychiatry.

[33] Florian M., Skurková L., Mesarčová L., Slivková M., Kottferová J. (2024). Decision-Making and Moral Distress in Veterinary Practice: What Can Be Done to Optimize Welfare Within the Veterinary Profession?. J. Vet. Med. Educ..

[34] Morgan C.A., McDonald M. (2007). Ethical Dilemmas in Veterinary Medicine. Vet. Clin. N. Am. Small Anim. Pract..

[35] Batchelor C.E.M., McKeegan D.E.F. (2012). Survey of the Frequency and Perceived Stressfulness of Ethical Dilemmas Encountered in UK Veterinary Practice. Vet. Rec..

[36] Martin F., Taunton A. (2006). Perceived Importance and Integration of the Human-Animal Bond in Private Veterinary Practice. J. Am. Vet. Med. Assoc..

[37] Springer S., Grimm H., Sandøe P., Lund T.B., Kristensen A.T., Corr S.A. (2022). Compete or Cooperate with ‘Dr. Google’? Small Animal Veterinarians’ Attitudes towards Clients’ Use of Internet Resources—A Comparative Study across Austria, Denmark and the UK. Animals.

[38] Robertson S. (2012). “Convenience” Euthanasia—A Comment. Can. Vet. J..

[39] Rathwell-Deault D., Godard B., Frank D., Doizé B. (2017). Conceptualization of Convenience Euthanasia as an Ethical Dilemma for Veterinarians in Quebec. Can. Vet. J..

[40] Hitchcock M., Workman M.K., Guthrie A.P., Ruple A., Feuerbacher E.N. (2024). Factors Associated with Behavioral Euthanasia in Pet Dogs. Front. Vet. Sci..

[41] McVety D. (2017). Convenience and Aggressive Pet Euthanasia. Treatment and Care of the Geriatric Veterinary Patient.

[42] Welsh L. (2015). The Euthanasia of Aggressive Dogs. Vet. Nurse.

[43] Mills D.S., Zulch H., Meints K., Shepherd K., Mannion C.J. (2013). Euthanasia of Healthy but Aggressive Dogs. Vet. Nurs. J..

[44] Siracusa C., Provoost L., Reisner I.R. (2017). Dog- and Owner-Related Risk Factors for Consideration of Euthanasia or Rehoming before a Referral Behavioral Consultation and for Euthanizing or Rehoming the Dog after the Consultation. J. Vet. Behav..

[45] Bubeck M., Springer S. (2024). Sociologising Veterinary Ethics: An Exploration of Professional Identity. EurSafe2024 Proceedings.

[46] Shaw J.R., Lagoni L. (2007). End-of-Life Communication in Veterinary Medicine: Delivering Bad News and Euthanasia Decision Making. Vet. Clin. N. Am. Small Anim. Pract..

[47] Pepper B.M., Chan H., Ward M.P., Quain A. (2023). Euthanasia of Dogs by Australian Veterinarians: A Survey of Current Practices. Vet. Sci..

[48] Kogan L.R., Rishniw M. (2023). Veterinarians and Moral Distress. J. Am. Vet. Med. Assoc..

[49] Official Gazette of the Republic of Turkey (2024). Law No. 5199 on the Protection of Animals. https://www.mevzuat.gov.tr/mevzuat?MevzuatNo=5199&MevzuatTur=1&MevzuatTertip=5.

[50] Official Gazette of the Republic of Turkey (2024). Law No. 7527 on the Amendment of the Law on the Protection of Animals. https://www.resmigazete.gov.tr/eskiler/2024/08/20240802-5.htm.

[51] Toksabay E. (2024). Turkey Passes Law to Round Up Stray Dogs, Opposition Vows Appeal, Reuters. https://www.reuters.com/world/middle-east/turkey-passes-law-get-stray-dogs-off-streets-into-shelters-2024-07-30/.

[52] Euronews (2024). “This Spells Death”: Inside Turkey’s New Bill That Could See Millions of Stray Dogs Killed. Euronews.

[53] Çınar B., Polat E., İspir Ç.B. (2025). Affective Polarization in Online Reactions to Turkey’s 2024 Stray Animal Law. OPUS Toplum. Araştırmaları Derg..

[54] Türkiye Barolar Birliği (2024). Hayvanları Koruma Kanunu’nda Değişiklik Yapılmasına Dair Kanun Teklifine İlişkin Hukuki Değerlendirme. Türkiye Barolar Birliği.

[55] Members of the Panel on Euthanasia, AVMA Staff Consultants (2020). American Veterinary Medical Association AVMA Guidelines for the Euthanasia of Animals 2020.

[56] World Small Animal Veterinary Association (2022). WSAVA Global Guidelines for the Euthanasia of Companion Animals 2022.

[57] Horecka K., Neal S. (2022). Critical Problems for Research in Animal Sheltering, a Conceptual Analysis. Front. Vet. Sci..

[58] Braam D.H., Bukachi S.A., Leiva D., Tasker A., Boden L., Bardosh K. (2025). Perspectives on the Social Sciences in Global Animal Health Governance: A Qualitative Study of Experts. Prev. Vet. Med..

[59] Papavasili T., Kontogeorgos A., Mavrommati A., Sossidou E.N., Chatzitheodoridis F. (2024). Review of Stray Dog Management: Dog Days in the European Countries. Bulg. J. Vet. Med..

[60] Ortega-Pacheco A., Jimenez-Coello M., Kuře J. (2011). Debate For and Against Euthanasia in the Control of Dog Populations. Euthanasia—The “Good Death” Controversy in Humans and Animals.

[61] Guilloux A.G.A., Panachão L.I., Alves A.J.S., Zetun C.B., Cassenote A.J.F., Dias R.A. (2018). Stray Dogs in Urban Fragments: Relation between Population’s Perception of Their Presence and Socio-Demographic Factors. Pesqui. Veterinária Bras..

[62] Animal Action Greece (2024). Greek Legislation. https://www.animalactiongreece.org/advice-and-welfare/greek-legislation/.

[63] (2012). Greek Law 4039/2012. On the Protection of Companion Animals and Stray Management. https://www.digihome.eu/law-number-40392012-government-gazette-a-15-for-home-pets-and-stray-pets-and-to-protect-animals-from-exploitation-or-use-for-profit/.

[64] Siettou C., Theodoropoulou E., Siettou A.S. (2024). An Overview of Greece’s Newly Established Progressive Stray Dog Management Laws. Pets.

[65] Hartnack S., Springer S., Pittavino M., Grimm H. (2016). Attitudes of Austrian Veterinarians towards Euthanasia in Small Animal Practice: Impacts of Age and Gender on Views on Euthanasia. BMC Vet. Res..

[66] Hannah D.R., Robertson K. (2020). Emotional Regulation in Veterinary Work: Do You Know Your Comfort Zone?. Can. Vet. J..

[67] Tannenbaum J. (1985). Ethics and Human-Companion Animal Interaction. Vet. Clin. N. Am. Small Anim. Pract..

[68] Ashall V. (2023). Reducing Moral Stress in Veterinary Teams? Evaluating the Use of Ethical Discussion Groups in Charity Veterinary Hospitals. Animals.

[69] Mullan S.M. (2012). Ethical Decision-making in Veterinary Practice: Using the Head and the Heart. Vet. Rec..

[70] Braun V., Clarke V. (2006). Using Thematic Analysis in Psychology. Qual. Res. Psychol..

[71] Braun V., Clarke V. (2019). Reflecting on Reflexive Thematic Analysis. Qual. Res. Sport. Exerc. Health.

[72] Braun V., Clarke V. (2021). One Size Fits All? What Counts as Quality Practice in (Reflexive) Thematic Analysis?. Qual. Res. Psychol..

[73] Braun V., Clarke V. (2022). Conceptual and Design Thinking for Thematic Analysis. Qual. Psychol..

[74] Charmaz K. (2013). Constructing Grounded Theory: A Practical Guide through Qualitative Analysis.

[75] Montoya A., Matthew S., McArthur M., Jarden A. (2025). Moral Conflict and Moral Distress in Veterinarians: A Mixed-methods Approach. Aust. Vet. J..

[76] Montoya A.I.A., Hazel S., Matthew S.M., McArthur M.L. (2019). Moral Distress in Veterinarians. Vet. Rec..

[77] Grimm H., Bergadano A., Musk G.C., Otto K., Taylor P.M., Duncan J.C. (2018). Drawing the Line in Clinical Treatment of Companion Animals: Recommendations from an Ethics Working Party. Vet. Rec..

[78] Ives J., Draper H. (2009). Appropriate Methodologies for Empirical Bioethics: It’s All Relative. Bioethics.

[79] Ziebland S., McPherson A. (2006). Making Sense of Qualitative Data Analysis: An Introduction with Illustrations from DIPEx (Personal Experiences of Health and Illness). Med. Educ..

[80] Kipperman B.S., Kass P.H., Rishniw M. (2017). Factors That Influence Small Animal Veterinarians’ Opinions and Actions Regarding Cost of Care and Effects of Economic Limitations on Patient Care and Outcome and Professional Career Satisfaction and Burnout. J. Am. Vet. Med. Assoc..

[81] Cornell K.K., Kopcha M. (2007). Client-Veterinarian Communication: Skills for Client Centered Dialogue and Shared Decision Making. Vet. Clin. N. Am. Small Anim. Pract..

[82] Charles N., Davies C.A. (2008). My Family and Other Animals: Pets as Kin. Sociol. Res. Online.

[83] Rock M., Mykhalovskiy E., Schlich T. (2007). People, Other Animals and Health Knowledges: Towards a Research Agenda. Soc. Sci. Med..

[84] Charmaz K. (2017). Constructivist Grounded Theory. J. Posit. Psychol..

[85] Carmichael T., Cunningham N. (2017). Theoretical Data Collection and Data Analysis with Gerunds in a Constructivist Grounded Theory Study. Electron. J. Bus. Res. Methods.

[86] Lu H., Hodge W.A. (2019). Toward Multi-Dimensional and Developmental Notion of Researcher Positionality. Qual. Res. J..

[87] Palinkas L.A., Horwitz S.M., Green C.A., Wisdom J.P., Duan N., Hoagwood K. (2015). Purposeful Sampling for Qualitative Data Collection and Analysis in Mixed Method Implementation Research. Adm. Policy Ment. Health Ment. Health Serv. Res..

[88] Malterud K., Siersma V.D., Guassora A.D. (2016). Sample Size in Qualitative Interview Studies. Qual. Health Res..

[89] Braun V., Clarke V., Terry G., Hayfield N., Liamputtong P. (2019). Thematic Analysis. Handbook of Research Methods in Health and Social Sciences.

[90] Richards L., Coghlan S., Delany C. (2020). “I Had No Idea That Other People in the World Thought Differently to Me”: Ethical Challenges in Small Animal Veterinary Practice and Implications for Ethics Support and Education. J. Vet. Med. Educ..

[91] Platt B., Hawton K., Simkin S., Mellanby R.J. (2012). Suicidal Behaviour and Psychosocial Problems in Veterinary Surgeons: A Systematic Review. Soc. Psychiatry Psychiatr. Epidemiol..

[92] Byrne D. (2022). A Worked Example of Braun and Clarke’s Approach to Reflexive Thematic Analysis. Qual. Quant..

[93] Finlay L. (2002). “Outing” the Researcher: The Provenance, Process, and Practice of Reflexivity. Qual. Health Res..

[94] Allsop D.B., Chelladurai J.M., Kimball E.R., Marks L.D., Hendricks J.J. (2022). Qualitative Methods with Nvivo Software: A Practical Guide for Analyzing Qualitative Data. Psych.

[95] Berger R. (2015). Now I See It, Now I Don’t: Researcher’s Position and Reflexivity in Qualitative Research. Qual. Res..

[96] Christiansen S.B., Kristensen A.T., Sandøe P., Lassen J. (2013). Looking after Chronically Ill Dogs: Impacts on the Caregiver’s Life. Anthrozoos.

[97] Bishop G., Cooney K., Cox S., Downing R., Mitchener K., Shanan A., Soares N., Stevens B., Wynn T. (2016). 2016 AAHA/IAAHPC End-of-Life Care Guidelines. J. Am. Anim. Hosp. Assoc..

[98] Leary S., Underwood W., Anthony R., Cartner S., Grandin T., Greenacre C., Gwaltney-Brant S., McCrackin M.A., Meyer R., Miller D. (2020). AVMA Guidelines for the Euthanasia of Animals.

[99] Dalla Villa P., Kahn S., Stuardo L., Iannetti L., Di Nardo A., Serpell J.A. (2010). Free-Roaming Dog Control among OIE-Member Countries. Prev. Vet. Med..

[100] Davlin S.L., VonVille H.M. (2012). Canine Rabies Vaccination and Domestic Dog Population Characteristics in the Developing World: A Systematic Review. Vaccine.

[101] Massei G., Fooks A.R., Horton D.L., Callaby R., Sharma K., Dhakal I.P., Dahal U. (2017). Free-Roaming Dogs in Nepal: Demographics, Health and Public Knowledge, Attitudes and Practices. Zoonoses Public. Health.

[102] Totton S.C., Wandeler A.I., Zinsstag J., Bauch C.T., Ribble C.S., Rosatte R.C., McEwen S.A. (2010). Stray Dog Population Demographics in Jodhpur, India Following a Population Control/Rabies Vaccination Program. Prev. Vet. Med..

[103] Smith L.M., Goold C., Quinnell R.J., Munteanu A.M., Hartmann S., Dalla Villa P., Collins L.M. (2022). Population Dynamics of Free-Roaming Dogs in Two European Regions and Implications for Population Control. PLoS ONE.

[104] Linder E., Grimm H. (2023). What Is Wrong with Eating Pets? Wittgensteinian Animal Ethics and Its Need for Empirical Data. Animals.

[105] Arluke A., Irvine L. (2017). Physical Cruelty of Companion Animals. The Palgrave International Handbook of Animal Abuse Studies.

[106] Springer S., Grimm H. (2022). The Future of Veterinary Ethics. Ethics in Veterinary Practice.

